# Retroperitoneal pararenal isolated neurofibroma: report of a case and review of literature

**DOI:** 10.3332/ecancer.2012.253

**Published:** 2012-05-15

**Authors:** C Corbellini, A Vingiani, F Maffini, A Chiappa, E Bertani, B Andreoni

**Affiliations:** 1 Division of General and Laparoscopic Surgery, European Institute of Oncology, University of Milan, 435 Ripamonti St., Milan 20141, Italy; 2 Pathological Division, European Institute of Oncology, University of Milan, Milan 20141, Italy

**Keywords:** *neurofibroma*, *rare tumour*, *benign tumour*, *kidney*, *retroperitoneal tumour*

## Abstract

The neurofibroma is a tumour of neural origin. This kind of neoplasm, though, is generally skin located. Rare cases in deep organs or in the peritoneal cavity are also reported in the literature. There are two types of neurofibromas, localized and diffuse; the latter is associated with von Recklinghausen disease and always occurs together with skin neurofibromas. Here we report the case of a 47-year-old man affected by retroperitoneal neurofibroma, but not associated with von Recklinghausen disease. A computed tomography (CT) scan described a retroperitoneal pararenal lesion with no clear involvement of adjacent viscera. We describe the diagnostic modality, treatment planning and the timing of treatment of this neoplasm, reviewing also the literature.

## List of abbreviations

US: ultrasonography
CT: computed tomography
CEA: carcinoembryonic antigen
CA19.9: carbohydrate antigen 19.9
FNB: CT-guided fine needle biopsy
ICV: inferior cava vein
RRV: right renal vein
Neu: neurofibroma
MRI: magnetic resonance imaging

## Clinical case

**Figure 1:**
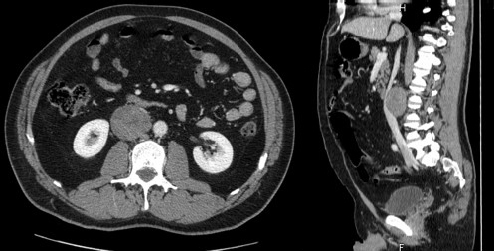
CT scan

A 47-year-old man presented at our attention for fever and abdominal pain in the right lumbar region without urinary symptoms. His medical history did not reveal any diseases. An abdominal ultrasonography detected an oval mass measuring 6 cm in diameter to the right para-aortic region. A CT confirmed the presence of a solid mass (53.3 × 48 × 60 mm^3^); the involvement of adjacent viscera was unclear, in particular the kidneys, renal vessels and right psoas muscle, while the cava vein appeared displaced in an anterior-medial direction (Figure 1). The mass appeared well-encapsulated and defined. No signs of von Recklinghausen’s disease were identified. Liver function tests were normal and preoperative tumour markers, including carcinoembryonic antigen (CEA) and carbohydrate antigen 19.9 (CA19.9), were not elevated.

**Figure 2:**
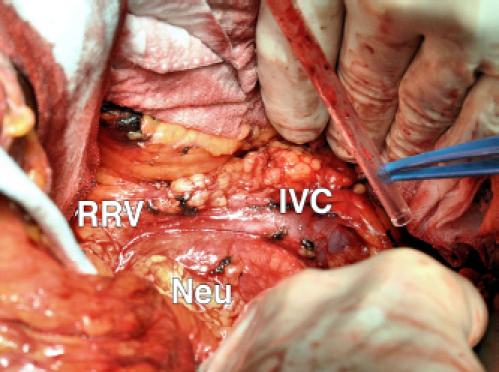
ICV: inferior cava vein; RRV: right renal vein; and Neu: Neurofibroma

**Figure 3:**
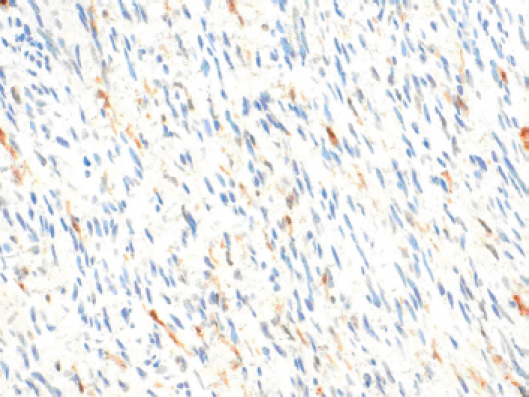
The expression of calretinin in our case, showing a weak stain in less than 25% of tumour cells, confirming the diagnosis of Neurofibroma

A CT-guided fine needle biopsy (FNB) showed a benign neoplasm of peripheral nerves tissue, characterized by the presence of elongated and wavy cells positive of S-100 protein. For these symptoms, the patient underwent a surgical resection that started with a subcostal incision after right ureteral stent placement. At the intra-abdominal exploration, the lesion appeared to constrict the cava vein and displace the right kidney, the renal vein (Figure 2) and the right ureter. These structures were identified and preserved after a sharp dissection. The specimen was extracted with en-bloc resection. Total operative time was 177 minutes with negligible intraoperative blood loss. The postoperative hospital course was uneventful, and the patient was discharged after 6 days. Pathology examination revealed a well-circumscribed lesion composed of loosely arranged tumour cells with the typical fascicular and wavy pattern of growth, plenty of collagen fibers and myxoid areas. We evaluated the expression of calretinin in our case, showing a weak stain in less than 25% of tumour cells, confirming the  diagnosis of Neurofibroma (Figure 3). During a multidisciplinary meeting, a clinical and instrumental follow-up was recommended. At  the 8-month follow-up, the CT scan was completely negative.

## Discussion

Neurofibromas, in general, are rare neoplasm and arise in patients with von Recklinghausen disease, but a solitary variant has been observed in rare cases and its splanchnic location is very uncommon [1].

Paraaortic–Pararenal Neurofibroma is an exceedingly rare tumour location [2]. To our knowledge, only six such cases have been reported worldwide to date (Table 1). Although their diagnosis and location is similar to that of Neurofibroma, different diagnostic and therapeutic approaches (surgery) have been used. In all cases, except ours and another one, patients underwent radical nephrectomy. In all likelihood, such an approach is due to the fact that preoperative imaging staging does not often allow one to diagnose these neoplasias with certainty.

As a matter of fact, only histology can diagnose them and, if it is performed preoperatively, it can influence treatment. In particular, it is capital to discriminate between malignant and benign lesions thus modifying a surgical approach, conservative versus aggressive ones. In  our case, the absence of mitotic activity, lack of necrosis, pleomorphism and infiltrative pattern of growth allow us exclude malignity.

Some authors say that using perfusion imaging may help differentiate renal parapelvic neurofibroma from the malignant lesions of the kidney [8].

In our case, the renal parenchyma and pelvicalyceal system were seen intraoperatively to be uninvolved. Because of the specific radiological results, in order to preoperatively plan the best strategy for the patients, we performed a percutaneous CT-guided biopsy, which, while feasible, could be deceptive [9]. Based on the pathological report and on the above-mentioned intraoperative findings, we decided to perform a total excision only of the lesion. From a literature review, it emerges that the treatment of choice for neurofibromas, in particular for solitary ones, is still controversial. Some authors think that the best treatment for solitary neurofibromas is radical nephrectomy [2]. Other authors posit that surgical resection is indicated only when the tumour causes pain or progressive neurological deficiencies, as  well as when there is a strong suspicion of malignancy [10]. Solitary Neurofibromas are associated with a low local recurrence rate, if completely excised [11].

## Conclusion

Planning the best treatment based on clinical and radiological data is often impossible. The most common risk for surgeons is to put the patient through a demolitive surgery, often an unnecessary surgical intervention. Preoperative histological diagnosis can be a useful tool to  help the surgeon choose demolitive surgery not only in selected cases but also in cases where the therapeutical implications are conservative or demolitive.

## Figures and Tables

**Table 1:** Six cases of paraaortic–pararenal neurofibroma

Case	Age	Sex	Imaging	Location	Surgery procedure
Freund et al (1967) [3]	45	F	Angiography, excretory urogram	Left lower pole calyces and renal pelvis	Local tumour excision
Borrego et al (1995) [4]	41	NA	CT, US, excretory urogram	Left renal sinus	Nephrectomy
Nishiyama et al (2000) [5]	33	F	CT	Right renal sinus	Retroperitoneoscopic tumour resection
Kostakopoulos et al (2003) [2]	37	F	CT	Right renal sinus	Nephrectomy
Eljack et al (2010) [6]	59	M	CT, MRI	Left renal sinus	Nephrectomy
Mondal et al (2010) [7]	54	F	CT, MRI	Right upper pole calyces	Nephrectomy
Our case	47	M	US, CT-biopsy	Right renal sinus	Local tumour excision
